# Draft Genome Sequence of *Lactobacillus plantarum* XJ25 Isolated from Chinese Red Wine

**DOI:** 10.1128/genomeA.01216-16

**Published:** 2016-11-17

**Authors:** Meijing Zhao, Shuwen Liu, Ling He, Yu Tian

**Affiliations:** a College of Enology, Northwest A&F University, Yangling, China; b Heyang Experimental and Demonstrational Stations for Grape, Weinan, Shaanxi, China; c College of Horticulture, Northwest A&F University, Yangling, China

## Abstract

Here, we present the draft genome sequence of *Lactobacillus plantarum* XJ25, isolated from Chinese red wine that had undergone spontaneous malolactic fermentation, which consists of 25 contigs and is 3,218,018 bp long.

## GENOME ANNOUNCEMENT

*Lactobacillus plantarum* can survive harsh winemaking conditions to efficiently conduct malolactic fermentation (MLF) (1, 2). Moreover, *L. plantarum* has been shown to produce bateriocins that could combat spoilage lactic acid bacteria in wine (3). And *L. plantarum* XJ25 was characterized as a bacteriocin-producing strain when it was cocultured with other lactic acid bacteria. Intrigued by the unique features of *L. plantarum* XJ25, we sequenced its genome to reveal its genetic structure and to explore its potential to be used as a novel MLF starter culture.

The genome of *L. plantarum* XJ25 was sequenced using an Illumina HiSeq 4000 platform. A paired-end library was constructed with an insert size of 350 bp. The filtered reads (1,364 Mb) were assembled by SOAPdenovo (4, 5) to generate 25 contigs and 24 scaffolds with approximately 400-fold coverage. Moreover, Gene prediction was performed using GeneMarkS (6). Coding genes were subsequently annotated with six databases, including KEGG (7), COG (8), NR, Swiss-Prot (9), and GO (10). With the COG database, a total of 2,323 coding sequences (CDSs) were divided into 21 functional groups.

*L. plantarum* XJ25 has a genome with an approximate size of about 3,218,018 bp, a mean GC content of 44.5%, and 3,075 CDSs; 58 tRNA genes were predicted with tRNAscan-SE (11). Furthermore, 62 transposons and 96 tandem repeats were identified. Moreover, the genome sequencing results for *L. plantarum* XJ25 showed the genetic potential for the production of metabolites. Specifically, a complete organization of the plantaricin locus was found in the XJ25 strain. Several single-nucleotide polymorphisms were identified in the function genes *pln*F, *pln*J, and *pln*K, which may lead to variations in the strain’s antimicrobial activity.

### Accession number(s).

This whole-genome shotgun project has been deposited at DDBJ/ENA/GenBank under the accession number MAXE00000000. The version described in this paper is the first version, MAXE01000000.
